# Asynchronous Broadcasting of Audiovisual Content as a Telerehabilitation Strategy for Patients in Rural Areas: Development and Usability Study

**DOI:** 10.2196/66343

**Published:** 2026-05-29

**Authors:** Lilia Aparicio Pico, Roberto Ferro Escobar, Paulo César Coronado Sánchez

**Affiliations:** 1 Faculty of Engineering, Universidad Distrital Francisco José De Caldas, Carrera 7 # 40B - 53, Bogotá DC, 110231, Colombia, 57 6013239300

**Keywords:** telerehabilitation, asynchronous, rehabilitation, telemedicine, telehealth, telebroadcasting, virtual therapy, video broadcasting, audiovisual, broadcasting, broadcast, case study, physical therapy, physiotherapy, occupational therapy, speech therapy, model, rural, access, accessibility, service, delivery

## Abstract

**Background:**

Geographical and economic barriers limit access to health care services in rural regions of Colombia. In San Vicente del Caguán, the lack of infrastructure and rehabilitation professionals forces patients to travel long distances. Asynchronous telerehabilitation using video broadcasting is a viable strategy to address these challenges.

**Objective:**

This study aims to design and validate a telerehabilitation model using asynchronous audiovisual content broadcasting for rural patients, evaluating functionality, usability, and clinical effectiveness.

**Methods:**

A 4-stage case study developed and validated the model in San Vicente del Caguán: (1) analysis of telemedicine experiences and video-based therapy; (2) solution design including telecommunications infrastructure (radio links and Wi-Fi), mobile app (HSRehabiAPP), and web platform (HSRehabiWEB); (3) fieldwork with 7 patients receiving physical, occupational, or speech therapy, evaluating functionality (11 criteria), usability (8 criteria), and content quality (5 criteria); and (4) results analysis. The infrastructure connected San Rafael Hospital with remote centers in Los Pozos and Tres Esquinas. Participants (aged 7-68 years) from urban and rural areas had conditions including stroke, shoulder injuries, knee pathologies, hypertension, and attention-deficit hyperactivity disorder.

**Results:**

All 7 patients achieved 100% compliance across functional, usability, and audiovisual content criteria. Functional evaluation covered login, navigation, therapy access, session viewing, exercise execution, pain assessment, therapist communication, and satisfaction surveys. Usability assessment evaluated initial access, content location, navigation comfort, instructional guidance, session organization, video playback, instruction clarity, and interface intuitiveness. Content criteria included exercise clarity, step-by-step instructions, visual quality, audio quality, and correct posture demonstration. Patients reported high satisfaction, noting reduced travel costs and time, family convenience, and effective outcomes. Offline functionality proved essential in areas with limited internet connectivity.

**Conclusions:**

The asynchronous audiovisual telerehabilitation model is an effective solution for improving access to rehabilitation services in rural areas. It successfully addressed geographical barriers and infrastructure limitations while maintaining clinical effectiveness across therapies. Implementation requires adequate technological infrastructure, user-friendly platforms with offline capabilities, and quality therapeutic content. Future work demands inclusive health policies, professional training, and research with larger sample sizes to assess long-term sustainability in diverse rural contexts.

## Introduction

Geographical barriers, population dispersion, and economic limitations hinder timely and continuous access to health care, especially in rural areas [1]. In addition, the lack of technological infrastructure and health care professionals forces patients to travel long distances to receive treatment [2]. In this context, telemedicine and telerehabilitation [3], which involve the use of information and communication technologies (ICT) to provide remote rehabilitation services [4], become particularly relevant in rural and hard-to-reach areas where health care resources are scarce [5].

A critical case in Colombia can be observed in San Vicente del Caguán, a municipality in the Department of Caquetá, where social conflict, coupled with difficult access and limited availability of physical, occupational, and speech therapy rehabilitation services, is exacerbated by the region’s geographical and topographical conditions. Although the San Rafael Hospital in the municipality offers rehabilitation services, it does not meet the demand from patients in rural areas. Consequently, the implementation of a telerehabilitation model based on the asynchronous broadcasting of audiovisual content emerges as a viable strategy to improve access to rehabilitation services and support the comprehensive recovery of patients facing geographical barriers. This proposal includes the use of interactive audiovisual content to facilitate patient education and remote monitoring as a solution for physical rehabilitation [6], while also training patients and their families, promoting self-management of health, and reducing dependence on in-person health care services [7]. The proposed infrastructure consists of a radio link and a mobile app containing interactive videos to asynchronously guide telerehabilitation patients.

## Methods

### Overview

The development of the model consists of 4 stages. The first stage is a case study analysis, aimed at identifying successful experiences to gather references on key aspects that impact the target groups served through telemedicine and telerehabilitation modalities. These aspects include patient satisfaction with the services provided, opportunities to improve access, cases of technological innovation, and technology-centered approaches with coverage analysis. In addition, important considerations regarding the creation and use of videos in therapy are taken into account. This stage also includes a specific case study focused on the regional conditions and services offered by San Rafael Hospital in San Vicente del Caguán and its surrounding areas, addressing telerehabilitation for physical therapy, occupational therapy, and speech therapy. The second stage focuses on designing the proposed solution to support the strategy’s model. The third stage involves fieldwork, including functional testing, usability testing, and audiovisual content evaluation, for which evaluation instruments were applied. Finally, the fourth stage involves analyzing the results to establish the final conclusions.

### Case Study Analysis

#### Case Analysis Focused on Service and Patients

Successful cases of telerehabilitation processes and medical consultations carried out through telemedicine platforms are presented in the study by Finak et al [8], highlighting the application of remote communication technologies for physical rehabilitation, occupational therapy, and chronic pain management [9], particularly for rural populations [10,11]. In addition, the following aspects were analyzed:

1. Patient satisfaction: the reviewed studies show high patient satisfaction with telemedicine and telerehabilitation interventions [12]. Similarly, rural patients reported high satisfaction, mainly due to reduced travel costs and time [13].
2. Access to care: telemedicine was found to be not only a viable but also a necessary solution to ensure equitable access to health care [14]. Other studies highlight improvements in the management of chronic diseases in rural areas, such as stroke management in China [15].
3. Technological innovation: the studies emphasize the innovative use of technologies such as mobile apps, eHealth platforms, and remote monitoring devices [16]. These tools enhance treatment adherence and chronic disease management. For example, improvements were reported in participation and self-efficacy among wheelchair users through an eHealth program [17].
4. Consistency measures: consistency across findings suggests that telemedicine and telerehabilitation technologies are effective across a variety of contexts and populations. Although the magnitude of the effects may vary, the general trend indicates improved accessibility, satisfaction, and clinical effectiveness [18].

#### Case Analysis Focused on Technologies

In this aspect, a review was conducted to understand how the type of technology used and the characteristics of the population influence the effectiveness of telemedicine and telerehabilitation interventions [19]. Effective components in different clinical contexts were analyzed, aiming to optimize technologies to maximize their benefits in medical practice [20].

Regarding access and coverage of telemedicine services in rural and remote areas, significant challenges were identified, which have been addressed through various telecommunication technologies. Key factors in choosing appropriate technology in contexts with limited communication infrastructure, such as rural areas [21], include the scarcity of terrestrial networks and the need for sustainable solutions. The use of technologies such as Very Small Aperture Terminals (VSAT) [22] is justified by their availability and adaptability to local needs, highlighting cost-effectiveness and broad coverage, especially in rural areas. Furthermore, in extremely remote locations such as Maewo Island [23], satellite technology emerges as the most viable solution [24].

For mobile networks in telemedicine, efforts focus on integrating patients and health care professionals through advanced ICT such as 5G, which offers high bandwidth and the capacity to quickly transmit large medical files [25], enabling timely and informed decision-making about patient health [26]. In addition, the future scalability of 5G and its ability to easily upgrade to new technologies are key advantages. The adaptation to available communication infrastructure, along with diverse technological options such as Code Division Multiple Access (CDMA 2000 1X) [27] and Global System for Mobile Communications (GSMC) [28,29], enhances availability and connection stability in both urban and rural environments, complemented by the use of ad hoc mobile networks such as Mobile Ad Hoc Networks (MANET) and Mobile Internet Protocol (Mobile IP) [30].

Worldwide Interoperability for Microwave Access (WiMAX) is another useful technology for covering large distances while providing adequate bandwidth for telemedicine applications, such as the transmission of high-resolution medical images and video [31].

Fiber optic infrastructure and radio networks also enhance access. In the study by Baena and Bríñez [32], a high-speed backbone network is shown to enable the creation of a faster and more robust telemedicine network, compared to other technologies such as satellite connections or radio links. In another case, radiofrequency-based solutions [33] connect a hospital with rural health posts in remote areas using a radio link.

In summary, the technologies used in telemedicine are diverse and vary according to the specific needs of each environment. WiMAX stands out for its high bandwidth and its ability to prioritize critical data. Studies presented in Niyato et al [34], Tchao et al [35], and Aliyu et al [36] further highlight these advantages. The use of portable devices and long-range communication protocols is also important. In the study by Pramanik et al [37], Long Range (LoRa) technology is selected for its broadband wireless access capabilities and security features, facilitating effective transmission of medical data.

Wireless Body Area Networks (WBAN) combined with cloud computing enable remote patient monitoring and real-time health data analysis. An example of remote health monitoring can be found in Suleiman and Adinoyi [38].

A description of how 5G technology, artificial intelligence, machine learning, and the Internet of Medical Things (IoMT) are transforming health care delivery is provided in the study by Latha et al [39]. In addition, WBAN technology [40] and cloud computing are used to create a telemedicine framework, as shown in the study by Park et al [40]. Finally, LTE-Advanced (LTE-A) can also be selected for its higher bandwidth, lower latency, better coverage, increased capacity, and improved quality of service, as highlighted in Kress and Leeuwen [41].

#### Application Case in San Vicente Del Caguán

To analyze the health care access issues in this region of Colombia, it is essential to consider the years of armed conflict experienced in the Department of Caquetá. This context has hindered progress in various sectors, such as infrastructure, education, and health, limiting access to essential basic services. These deficiencies, recognized as unsatisfied basic needs, not only contradict the Sustainable Development Goals but also widen socioeconomic and territorial inequality gaps in the region.

In the Department of Caquetá, medium-complexity health services are centralized in Florencia, the department’s capital. In some municipalities, specialist services (such as gynecology, cardiology, internal medicine, and pediatrics) are only offered through mobile health brigades. Outside the capital, municipalities only provide low-complexity health services, which results in an uneven distribution of specialists across the department. In addition, there is insufficient supply, high demand, long waiting times for appointments managed by health entities, and geographical limitations.

The San Rafael Hospital in San Vicente del Caguán has recently opened a Comprehensive Rehabilitation Center, offering physical therapy, occupational therapy, and speech therapy, making it the only municipality outside Florencia with these 3 rehabilitation services available.

San Vicente del Caguán covers an area of 28,300 km² and has a population of approximately 54,575 inhabitants, where 51.3% of the population lives in rural areas, according to 2024 projections by Departamento Administrativo Nacional de Estadística [42]. The municipality is made up of 287 rural settlements distributed across 14 police inspection zones within the municipal territory. The primary access routes are dirt roads (unpaved), which significantly complicate the journey of rural patients traveling from their villages to San Rafael Hospital for various health care procedures.

In addition, there is no internet access at health service points in the region. However, a significant number of inhabitants own mobile phones, which they use for telephone calls and, when possible, access mobile data services in communal zones where coverage is available.

Considering these regional conditions, the proposed telerehabilitation system, based on broadcasting therapy content (physical therapy, occupational therapy, and speech therapy) through an asynchronous computer-based format, offers a viable solution to expand health care service coverage to rural areas served by San Rafael Hospital. This model would also overcome geographical barriers and internet access limitations, improving health care delivery in the region.

#### Design and Production of Audiovisual Content for Therapy

A video is an audiovisual content format that combines visual and audio elements to facilitate the understanding of a message and enhance information retention [43-45]. Videos can vary in format, duration, and purpose and are widely used across different industries, including education, entertainment, advertising, and health care, particularly in telerehabilitation where digital platforms have shown effectiveness [46,47].

Based on these criteria, teletherapy is developed as a care modality that uses ICTs, including mobile apps and video-based content, to deliver remote therapeutic rehabilitation services [46].

#### Considerations for the Use of Videos in Telerehabilitation

When supporting the use of videos in teletherapy, it is necessary to consider the following aspects: the positive impacts that are reflected in the improvement of the understanding of exercises, by providing a visual representation, allow patients to observe precise movements, postures, and appropriate techniques, which reduces the likelihood of errors and increases the accuracy in the execution of exercises [46]. In turn, adherence to rehabilitation treatment is another aspect that is strengthened with the use of videos.

Another significant impact is the reduction of the patients’ dependence on the therapist. Video-based interventions and visual feedback allow patients to perform exercises more independently, providing a constant guide that they can review as many times as necessary [48]. In Latin America, several studies have also demonstrated significant positive impacts. Video-based therapeutic interventions for rehabilitation resulted in significant improvements in mobility and pain reduction [49]. Technology-based rehabilitation interventions for poststroke patients helped improve motor coordination and reduce recovery time [50]. Technology-assisted rehabilitation programs have reported improvements in aerobic capacity and reduction of fatigue in patients with chronic conditions [51]. Rehabilitation programs have shown faster recoveries and pain reduction in postsurgical hip replacement patients [52].

The design of rehabilitation videos requires a methodical approach to ensure that audiovisual content is accurate, understandable, and accessible to all patients. Studies have highlighted [53] the importance of a systematic approach to creating educational videos for rehabilitation.

The need for a structured process for the design of audiovisual content has been emphasized in rehabilitation research [53], highlighting the importance of conducting pilot tests and collecting patient feedback to improve the quality of the videos, an iterative approach that makes it possible to adjust the content to make it more effective and accessible.

Finally, it is important to take into account that the methods or processes that guide the design of videos in telerehabilitation should consider the following recommendations: having the collaboration of multidisciplinary professionals [53], the duration of the video should be short enough to maintain the patient’s attention, but long enough to cover all important aspects of the exercise or technique [46], including clear and descriptive visual elements, as effective visual communication is essential in digital health interventions [54], to address the diversity of patients’ needs, the videos must be adaptable and customizable [51], the creation of videos must be an iterative process that includes evaluation and continuous feedback from patients and health care professionals, as demonstrated in recent systematic reviews [55], and it is important to consider options for downloading videos in areas with limited internet access [42].

### Development of the Telerehabilitation Model for the Implementation of the Content Broadcasting Strategy

#### Overview

The following are the 3 components of the model, consisting of the telecommunications infrastructure, the mobile app, and the design of audiovisual content with its mode of operation. The teletherapy services implemented support the telerehabilitation services for physiotherapy, occupational therapy, and speech therapy.

The solution has 3 components: a telecommunications component supported by a wireless network based on radiofrequency solutions (long-range and high-bandwidth radio links) and Wi-Fi technologies (802.11a, 802.11b, 800.11n, 802.11ac, and 802ax), which enable the interconnection of a hospital with prioritized and remote health posts far from the municipal or urban center. A software platform (Progressive Web Application [PWA]; Google), user-friendly and interactive, is complemented by the creation of audiovisual content (videos and animations) that are stored in a cloud repository or are synchronously and programmatically loaded with a rehabilitation therapy plan onto the patient’s mobile device. This solution can be available for direct access to the cloud or located at remote or rural centers for loading onto the patient’s mobile device to be used later at home. The platform allows the administration and configuration of therapies that will be scheduled for patients according to their medical history, needs, and clinical records.

#### The Implemented Network

**Figure 1.**
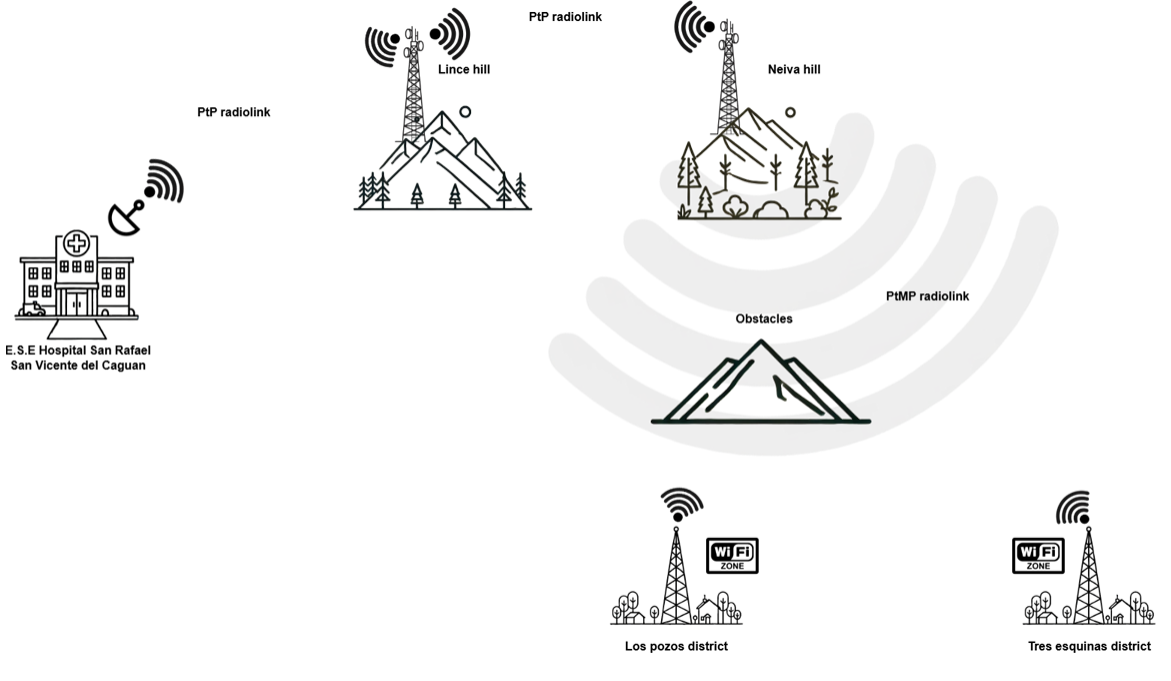
General configuration of the telecommunications system. (Own elaboration).

Figure 1 shows the general configuration of the telecommunications system.

The network is made up of a radio link that guarantees access to rehabilitation services in the telemedicine modality to connect the E.S.E. Hospital San Rafael with remote health centers with difficult access to serve the communities of Los Pozos and Tres Esquinas. The network is composed of several components described below.

E.S.E. Hospital San Rafael in San Vicente del Caguán: from this point, the telerehabilitation service is coordinated, bringing together specialists, training patients referred to teletherapy, and providing follow-up through tele-expertise to patients by connecting doctors and nurses through the radio link. These professionals are responsible for maintaining direct contact with patients once they start follow-up when they come to the center for their check-ups. Finally, monitoring is carried out via telemonitoring and synchronous and asynchronous teleconsultation.

The system has 2 signal relay hops at Lince Hill and Cerro Neiva. This configuration allows communication coverage to reach the remote areas.

**Figure 2.**
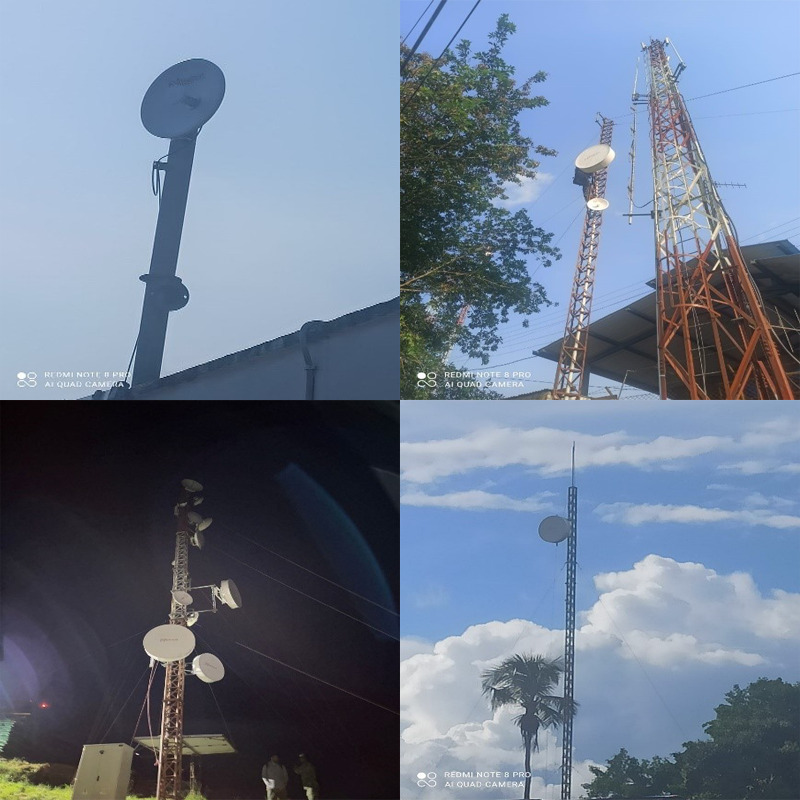
Installation of the system antennas.

Figure 2 shows the installed infrastructure at each of the described points. Figure 2A shows the antenna installed at Hospital San Rafael. Figure 2B presents the configuration at Lince Hill, which maintains a direct line with the hospital. Figure 2C shows the configuration at Cerro Neiva, which maintains a link with Lince Hill. From Cerro Neiva, the highest point in the configuration, communication is established with Tres Esquinas and Los Pozos, as shown in the configuration in Figure 2D for each point.

#### Implemented Software System

**Figure 3.**
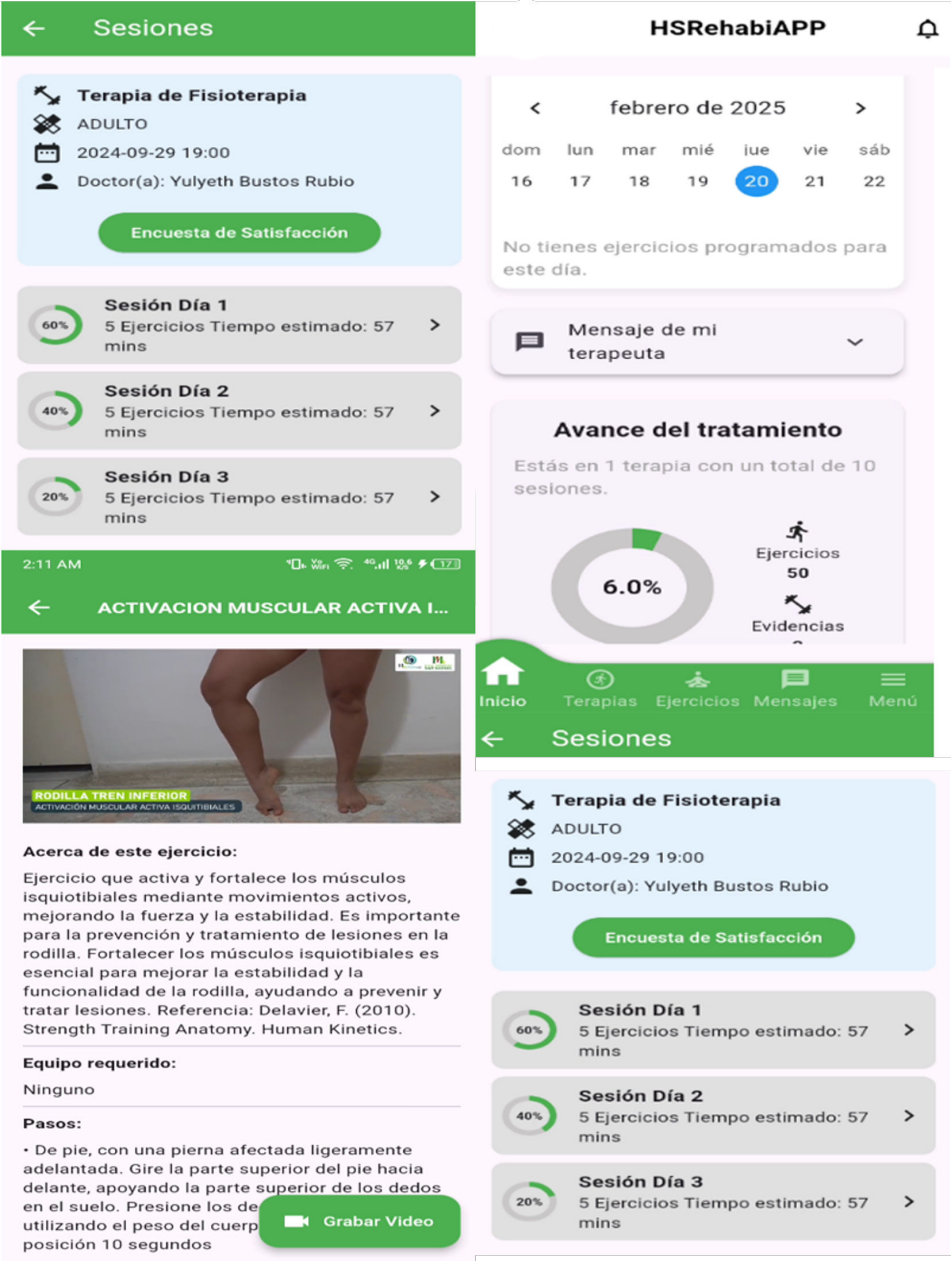
HSReHabiAPP mobile app.

The software system of the solution follows a layered architecture that ensures separation of responsibilities. The presentation layer consists of 2 apps. The first one, HSRehabiAPP, shown in Figure 3, is a mobile app designed for online and computer-based use, where users can access their assigned therapies, obtain exercise instructions, prepare evidence, monitor their progress, and securely communicate with the assigned health care professional.

A critical aspect in the design of the mobile app is its offline functionality. This is particularly important considering that most patients are in rural areas with little or no internet connectivity. Data synchronization is fully automated and takes place whenever patients have a connection, using a publish-subscribe model that optimizes data transfer.

**Figure 4.**
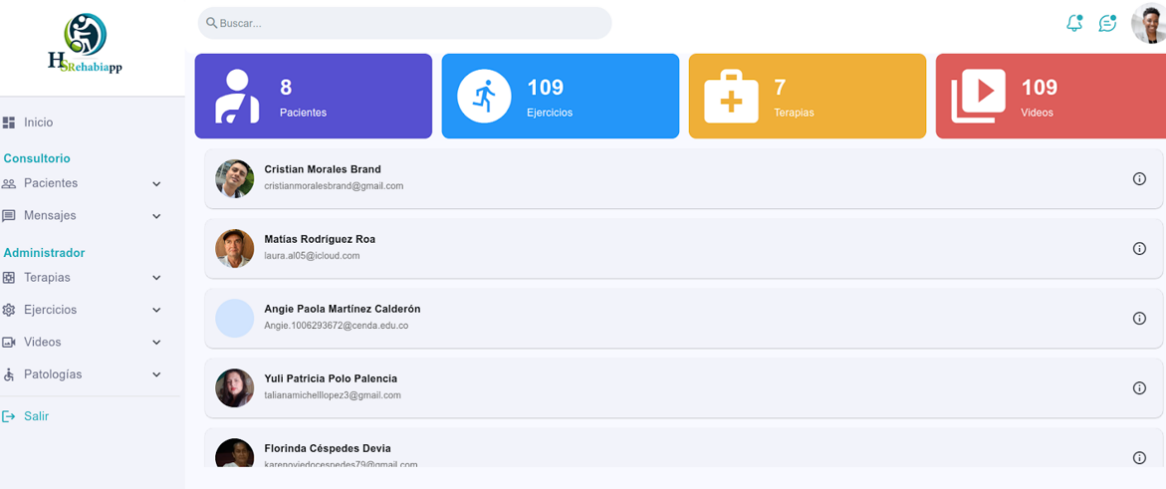
Main page of HSRehabiWEB.

The second app, HSRehabiWEB (Figure 4), is a web app where health care professionals manage the catalog of videos and exercises, configure therapies, register their patients, and monitor their progress. This app serves as the control center of the solution.

The application layer consists of an application programming interface, which provides access points to the application logic (Backend). The application layer is deployed on the AWS platform (Amazon) to ensure adequate quality of service. The data layer, also deployed on AWS, implements a relational model in a PostgreSQL database (Amazon).

### Field Work

#### Overview

A pilot test was carried out between the San Rafael Hospital in San Vicente del Caguán and the centers of Pocos and Tres Esquinas in rural areas, to evaluate the implementation and effectiveness of the telerehabilitation model based on asynchronous transmission of audiovisual content in the municipality of San Vicente del Caguán, Colombia. The test included 7 patients in the telerehabilitation program, which covered physical, occupational, and speech therapies. The criteria observed considered functional tests, usability criteria, and content evaluation.

#### Description of Participants

Table 1 summarizes the demographic, clinical, and geographic characteristics of the patients in the trial.

**Table 1.** Demographic, clinical, and geographic characteristics of the patients in the pilot test of the telerehabilitation model.

ID	Name	Area	Pathology	Life cycle	Age (y)	Zone	Disability	Surgeries
P-1	Cristian Iván Morales Brand	Physical therapy	Left shoulder dislocation	Adult	25	Urban	No	Shoulder surgery
P-2	Matías Rodríguez Roa	Physical therapy	Stroke with left hemiplegia	Adult	55	Rural	Physical	Stroke
P-3	Yuli Patricia Polo Palencia	Physical therapy	Rotator cuff syndrome (right shoulder)	Adult	Not specified	Urban	No	No
P-4	Florinda Céspedes Debia	Speech therapy	Hypertension	Older adult	68	Rural	No	Hypertension
P-5	Emmanuel Lozano Ríos	Occupational therapy	Attention-deficit hyperactivity disorder	Early childhood	7	Rural	No	No
P-6	Jheisson Aguilera Betancourt	Physical therapy	Patella dislocation and right meniscus tear	Adult	31	Rural	No	Right knee surgery
P-7	Cristian Camilo Castro Cuéllar	Physical therapy	Knee sprain	Adult	27	Urban	No	No

#### Evaluation Instruments

Table 2 presents the criteria evaluated during the tests of the telerehabilitation model and its app, organized by number, specific criterion, and corresponding description. The evaluation covered 3 main areas: functional testing, usability testing, and audiovisual content assessment. Functional testing verified key aspects related to the usability, accessibility, and functionality of the app. Usability testing focused on identifying strengths and opportunities for improvement in the user experience by analyzing different aspects of user interaction with the app. Additionally, the audiovisual content evaluation examined the quality and effectiveness of the multimedia materials used in the telerehabilitation model to ensure that patients could perform the exercises properly and safely.

**Table 2.** Evaluation for CF^a^, CU^b^, and CA^c^ in the telerehabilitation model.

Number	Criterion	Description
CF-1	App login	Were you able to successfully log in using the email and password provided during registration?
CF-2	Navigation through the patient’s main page	Were you able to easily access and navigate through the patient’s main page, including the calendar, exercises, and messages sections?
CF-3	Access to the therapy module	Were you able to enter the therapy module and view the assigned therapies and their progress?
CF-4	Viewing scheduled therapy sessions	Were you able to access the scheduled sessions for a specific therapy and monitor their progress?
CF-5	Viewing exercises scheduled per session	Were you able to access the exercises defined for each session and track their progress?
CF-6	Execution of scheduled exercises	Were you able to see the instructions, illustrative videos, and other necessary details to correctly perform the exercises?
CF-7	Recording exercise execution	Does the app allow you to properly record and save the execution of an exercise?
CF-8	Pain assessment after the session	Were you able to enter and evaluate your pain level at the end of the session using a 0 to 10 pain scale?
CF-9	Communication with the therapist	Were you able to send messages to your therapist and receive responses through the message module?
CF-10	Therapy satisfaction assessment	Were you able to access and complete the satisfaction survey after completing the therapy sessions?
CF-11	Navigation through the side menu	Were you able to access additional features such as “My therapies,” “Messages,” and “Settings” through the side menu?
CU-1	Initial access	Was it easy for you to log into the app for the first time?
CU-2	Assigned content	Were you able to quickly find the videos of your assigned exercises?
CU-3	Navigation	Do you feel comfortable navigating through the app menus?
CU-4	Instructional guide	Did you find the step-by-step guide provided by the app at startup helpful?
CU-5	Session organization	Do you find the way your rehabilitation sessions are displayed clear?
CU-6	Video playback	Were you able to play the exercise videos without any issues?
CU-7	Therapist instructions	Was it easy for you to follow the therapist’s instructions through the app?
CU-8	Intuitive interface	Do you feel that the app’s interface is intuitive and easy to understand?
CA-1	Clarity in the presentation of the exercise	Do the videos clearly present the name and objective of the exercise?
CA-2	Step-by-step instructions	Do the videos adequately explain each step of the exercise in a way that is understandable for the patient?
CA-3	Visual quality	Is the video image quality sufficient to clearly observe the exercise movements?
CA-4	Audio quality	Is the audio in the videos clear, and is the verbal explanation of the exercise understandable without interference?
CA-5	Correct postures movements	Does the video highlight the correct postures and proper movements, showing how to perform the exercise safely?

a CF: functional criteria.

b CU: usability criteria.

c CA: audiovisual content criteria.

### Ethical Considerations

#### Ethics Approval

This study was approved by the Research Ethics Committee of Universidad Distrital Francisco José de Caldas. All procedures performed in this study involving human participants were conducted in accordance with the ethical standards of the institutional research committee and with the 1964 Helsinki Declaration and its subsequent amendments. Written informed consent was obtained from all participants included in the study (or from their legal guardians in the case of minors) prior to their participation. Participants were provided with detailed information regarding the nature and objectives of the telerehabilitation model, the procedures involved, the potential risks and benefits, and their right to withdraw from the study at any time without penalty. All participants were given sufficient time to consider their participation and had the opportunity to ask questions before signing the informed consent forms.

#### Privacy and Confidentiality

Patient data, including demographic information, medical records, therapy videos, and evaluation results, were securely stored in encrypted digital formats with access restricted exclusively to authorized members of the research team. The mobile app (HSRehabiAPP) and the web platform (HSRehabiWEB) were designed with security measures aimed at protecting personal health information, in compliance with Colombian data protection regulations (Law 1581 of 2012 on personal data protection). Video recordings used for therapy sessions were stored on secure servers and were accessible only to the assigned health care professionals and the corresponding patient. Participants were informed about data storage, access, and usage procedures through the informed consent process.

#### Vulnerable Populations

Additional ethical considerations were adopted to ensure adequate protection of these participants. In the case of the older adult participant, cognitive capacity to provide informed consent was assessed prior to enrollment in the study. For the minor participant, both parental informed consent and child assent were obtained, and therapy sessions were adapted according to the child’s age and conducted under parental supervision. Given the rural and potentially socioeconomically vulnerable condition of participants from Los Pozos and Tres Esquinas, measures were taken to ensure that participation was entirely voluntary and that individuals did not feel coerced to participate due to limited access to alternative rehabilitation services.

#### Data Sharing and Dissemination

Anonymized participant data will not be made publicly available due to the small sample size and the potential risk of reidentification, particularly considering the specific rural and clinical context of the participants. Aggregate results and findings are presented in this publication in a manner that protects the individual confidentiality of participants.

## Results

Table 3 presents the results obtained from the evaluation of the telerehabilitation model, including functional tests, usability tests, and audiovisual content assessment conducted with 7 patients (P-1 to P-7). The evaluated criteria are organized in rows, while the patients are presented in columns, indicating whether each criterion was met. Functional test results describe the performance of the telerehabilitation app according to the established functional criteria. Usability test results analyze the interaction of patients with the app, identifying the fulfillment of usability criteria (CU-1 to CU-8). Additionally, the audiovisual content evaluation presents the results associated with 5 assessment criteria (CA-1 to CA-5), focused on the quality and effectiveness of the multimedia materials used to support the correct and safe execution of rehabilitation exercises within the telerehabilitation model.

**Table 3.** Results for CF^a^, CU^b^, and CA^c^ by patient in the telerehabilitation model^d^.

Patient \ Criterion	P-1	P-2	P-3	P-4	P-5	P-6	P-7
CF-1	✔	✔	✔	✔	✔	✔	✔
CF-2	✔	✔	✔	✔	✔	✔	✔
CF-3	✔	✔	✔	✔	✔	✔	✔
CF-4	✔	✔	✔	✔	✔	✔	✔
CF-5	✔	✔	✔	✔	✔	✔	✔
CF-6	✔	✔	✔	✔	✔	✔	✔
CF-7	✔	✔	✔	✔	✔	✔	✔
CF-8	✔	✔	✔	✔	✔	✔	✔
CF-9	✔	✔	✔	✔	✔	✔	✔
CF-10	✔	✔	✔	✔	✔	✔	✔
CF-11	✔	✔	✔	✔	✔	✔	✔
CU-1	✔	✔	✔	✔	✔	✔	✔
CU-2	✔	✔	✔	✔	✔	✔	✔
CU-3	✔	✔	✔	✔	✔	✔	✔
CU-4	✔	✔	✔	✔	✔	✔	✔
CU-5	✔	✔	✔	✔	✔	✔	✔
CU-6	✔	✔	✔	✔	✔	✔	✔
CU-7	✔	✔	✔	✔	✔	✔	✔
CU-8	✔	✔	✔	✔	✔	✔	✔
CA-1	✔	✔	✔	✔	✔	✔	✔
CA-2	✔	✔	✔	✔	✔	✔	✔
CA-3	✔	✔	✔	✔	✔	✔	✔
CA-4	✔	✔	✔	✔	✔	✔	✔
CA-5	✔	✔	✔	✔	✔	✔	✔

a CF: functional criteria.

b CU: usability criteria.

c CA: audiovisual content criteria.

d Convention: ✔: Compliant

## Discussion

### Principal Findings

The considerations made based on the literature analysis to establish the system solution and the telerehabilitation strategy using audiovisual content broadcasting facilitated the validation of the proposal and verification through the case study in San Vicente del Caguán.

Regarding improved access to care, it was observed that telemedicine and telerehabilitation facilitate access to health care services, especially in regions with limited infrastructure.

In terms of patient satisfaction, participants expressed high satisfaction with these services, highlighting the timeliness of care and the convenience for families during recovery processes. In addition, patients noted a reduction in travel costs (both in time and money) and satisfactory levels of treatment effectiveness, achieving acceptable recovery outcomes across the 3 therapy types.

On the other hand, regarding the feasibility in rural health care centers, the technologies implemented proved effective in rural areas, where technology helps address the shortage of specialists to meet service demand. The ease of extending coverage was noted, improving the hospital’s service offering, while patients readily accepted and engaged with asynchronous computer-based therapies.

It was necessary to develop training and continuous education tools for all community members involved in the process. In addition, this initiative opened the opportunity to formulate inclusive health policies, recognizing the need to cover diverse conditions and populations, particularly those affected by economic and technological limitations.

Finally, the need for ongoing evaluation and monitoring was established to ensure the impact and effectiveness of technology in supporting patient recovery cases.

### Conclusions

The implementation of the telerehabilitation model based on the broadcasting of audiovisual content has proven to be a highly effective strategy for improving access to rehabilitation services in rural areas.

In the case study conducted in San Vicente del Caguán, patients showed high levels of satisfaction with the telerehabilitation interventions. The use of interactive audiovisual content not only facilitated rehabilitation in physical therapy, occupational therapy, and speech therapy, but also empowered patients and their families, promoting greater self-management of their health.

Findings from the literature review highlight the need to integrate telerehabilitation into public health policies, especially in rural contexts. Health authorities should develop appropriate technological infrastructure and promote continuous training for health care professionals to maximize the benefits of these technologies. Furthermore, inclusive policies should be implemented to ensure equitable access to telemedicine and telerehabilitation services for all populations, regardless of economic or technological limitations.

Several areas for future research were identified, including studies with larger sample sizes and long-term follow-up to assess the sustainability of the observed effects. It is also essential to explore the implementation of these technologies in low- and middle-income settings, as well as to investigate new technologies and innovative approaches that could further enhance the effectiveness and accessibility of telerehabilitation.

The positive effects of telemedicine and telerehabilitation interventions remain consistent across studies, regardless of the specific technology used or the target population. This indicates that the observed benefits are not specific to a particular context but are generalizable to various situations and populations.

Despite the variability in the evaluation methods used in the studies, including satisfaction surveys, clinical measures, and cost analyses, the overall findings remained robust. This suggests that telemedicine and telerehabilitation technologies are consistently effective, regardless of how outcomes are measured.

## Abbreviations

CDMA: Code Division Multiple Access
GSMC: Global System for Mobile Communications
ICT: information and communication technologies
IoMT: Internet of Medical Things
LoRa: Long Range
LTE-A: LTE-Advanced
MANET: Mobile Ad Hoc Networks
Mobile IP: Mobile Internet Protocol
PWA: Progressive Web Application
VSAT: Very Small Aperture Terminals
WBAN: Wireless Body Area Networks
WiMAX: Worldwide Interoperability for Microwave Access
